# Monoclonal antibodies associated IgA nephropathy or other nephropathies

**DOI:** 10.3389/fphar.2026.1891000

**Published:** 2026-09-30

**Authors:** Miłosz Miedziaszczyk, Gracjan Kosiorowski, Janusz Kubasik, Tomasz Grabowski, Edyta Szałek, Cheuk Chun Szeto, Ilona Idasiak-Piechocka

**Affiliations:** 1 Department of Clinical Pharmacy and Biopharmacy, Poznan University of Medical Sciences, Poznan, Poland; 2 Department of Inorganic Chemistry, Medical University of Gdansk, Gdansk, Poland; 3 Carol & Richard Yu Peritoneal Dialysis Research Centre, Prince of Wales Hospital, Department of Medicine & Therapeutics, The Chinese University of Hong Kong, Hong Kong, Hong Kong SAR, China; 4 Li Ka Shing Institute of Health Sciences (LiHS), Faculty of Medicine, The Chinese University of Hong Kong, Hong Kong, Hong Kong SAR, China; 5 Department of General and Transplant Surgery, Poznan University of Medical Sciences, Poznan, Poland

**Keywords:** adverse effect, glomerulonephritis, IgA nephropathy, mAb, monoclonal antibodies

## Abstract

The use of monoclonal antibodies (mAbs) has revolutionized the treatment of autoimmune, inflammatory, and neoplastic diseases. However, accumulating evidence indicates that biologic therapies may be associated with renal adverse events, including various types of glomerulonephritis. The aim of this review was to summarize all reports available in the scientific literature regarding the occurrence of IgA nephropathy after monoclonal antibody treatment. Additionally, the risk of other glomerulonephritis after biologic therapy was discussed. IgA nephropathy (IgAN) is the most commonly reported glomerular lesion associated with monoclonal antibody therapy, particularly with TNF-α inhibitors such as infliximab, adalimumab, golimumab, and etanercept, as well as with immune checkpoint inhibitors targeting the PD-1 (pembrolizumab, nivolumab) and interleukin (ustekinumab) pathways. Proposed mechanisms include immune dysregulation with a shift toward Th2 and Th17-dependent responses, increased production of galactose-deficient IgA1, complement activation, formation of circulating immune complexes, and loss of immune tolerance. In some cases, drug discontinuation and glucocorticoid therapy have resulted in partial or complete disease remission, although progression to end-stage renal disease has also been reported. Due to the potential severity of these complications, initial and periodic monitoring of renal function and urinalysis are recommended in patients receiving monoclonal antibody therapy. Early recognition of renal involvement and prompt treatment modification can improve prognosis.

## 1 Introduction

In recent years, we have observed significant progress in the development of biological therapies, among which monoclonal antibodies, including inhibitors of tumor necrosis factor alpha (TNF-α), occupy a special place. The registration of these drugs has opened up new therapeutic possibilities. Therapies based on monoclonal antibodies have revolutionized modern medicine, enabling effective inhibition of specific immune pathways and inhibiting disease progression and improving the prognosis in chronic diseases (Nelson et al., 2010; Chames et al., 2009), such as rheumatoid arthritis, psoriasis, or nonspecific bowel diseases (Tracey et al., 2008; Feldmann and Maini, 2003). However, the use of TNF-α inhibitors and other monoclonal antibodies is associated with adverse effects, both general (such as increased risk of infection or allergic reactions) and more specific (Bongartz et al., 2006). Increasingly, case reports appear in the literature indicating a possible relationship between biological therapy and the occurrence of renal complications, in particular glomerulonephritis. Particular attention is paid to cases of IgA nephropathy (IgAN) developing after the use of TNF-α inhibitors (Premuzic et al., 2020; Strobel et al., 2020). The mechanism of induction of this pathology in the context of biological therapy is not fully understood. It is assumed that it may include modulation of the immune response and disruption of immune tolerance. Cells in the kidney, including mesangial and endothelial cells, possess functional Fcγ receptors (FcγR) that can bind immune complexes and trigger inflammatory responses. Activation of FcγR on mesangial cells activates proinflammatory cells, primarily macrophages, and induces the expression of proinflammatory cytokines and profibrotic factors, contributing to kidney damage (Lopez-Parra et al., 2012). FcγRIII—and especially its soluble form FcγRIIIb—is markedly elevated in IgAN and is associated with disease severity (Li et al., 2022). The aim of this review is to summarize the current reports on the occurrence of glomerulonephritis after the use of monoclonal antibody therapy.

## 2 Monoclonal antibodies associated with the risk of IgA nephropathy

The mechanisms discussed below are based primarily on hypotheses proposed by the authors of individual case reports, experimental studies, and current knowledge of immunopathogenesis. Direct mechanistic evidence in patients with monoclonal antibody-associated glomerular disease is currently lacking, and these mechanisms should therefore be considered speculative.

### 2.1 Infliximab

Infliximab (INX) is a chimeric recombinant antibodyconsisting of a murine variable region and a human IgG1 constant region. It binds to both soluble and membrane-bound TNF-α, both soluble and membrane-bound, thereby inhibiting TNFα-mediated inflammatory signaling. In addition, infliximab has been reported to reduce the expression of adhesion molecules such as intercellular adhesion molecule 1 (ICAM-1) and vascular cell adhesion molecule 1 (VCAM-1). However, the relevance of these effects to the development of IgAN remains uncertain. (Jacquet et al., 2009). The described patient cases initially presented similar symptoms of IgAN. All patients experienced at least 2 of the 3 symptoms: hematuria, proteinuria, and deterioration of renal function.

Only in one case was a renal function test performed before initiating INX (no hematuria or proteinuria, estimated glomerular filtration rate (eGFR) 81.4 mL/min/1.73 m^2^ (Segawa et al., 2020). The most common adverse events occurred during drug infusion (dyspnea, urticaria, headache). Other adverse events included opportunistic infections, pancytopenia, hemolytic anemia, anaphylaxis, demyelinating diseases, interstitial pneumonia, pancreatitis, hepatitis, and IgAN (Shimizu et al., 2024). After the diagnosis of IgAN, INX treatment including angiotensin II receptor blockers (ARBs) (Jacquet et al., 2009; Kluger et al., 2015) was discontinued, and in another reported case (Segawa et al., 2020), due to poor control of psoriasis symptoms, glucocorticosteroids (GCS) were initially administered with average results, then switched to secukinumab, restoring control of the primary disease and IgAN. In the study presented by Nicolas Kluger et al. (Kluger et al., 2015), ustekinumab (UST) was used as a substitute for INX. Five years later, the first case of IgAN following UST treatment was published, describing the induction of IgAN after its use (a patient with Crohn’s disease), until then considered safe for the kidneys (Kanazawa et al., 2020). Montero SS et al. presented two cases of IgAN after successful long-term therapy of Crohn’s disease with infliximab. After 7 and 8 years, respectively, there was a deterioration of renal function (eGFR 36–51 mL/min/1.73 m^2^, proteinuria, hematuria), infliximab was discontinued, GCS and ARB were administered, achieving improvement of the above-mentioned disorders. In the first case, there was remission of Crohn’s disease, hence ustekinumab was included with positive results. In the second case, no change in therapy of the underlying disease was necessary (Montero et al., 2024).

Shimizu A et al. (Shimizu et al., 2024) presented 3 cases of Crohn’s disease treated with INX (2) and adalimumab in which IgAN developed after 3–6 years of treatment (patients had impaired renal function before starting INX,none of the patients underwent an initial kidney biopsy). After the occurrence ofIgAN, the authors did not discontinue INX, but started GCS therapy and performed tonsilectomy. Renal function improved, while Crohn’s disease remained controlled. The production of galactose-deficient IgA1 (Gd-IgA1) is mainly carried out by B lymphocytes and plasma cells, located mostly in the centers of the tonsils and lymph nodes. These observations suggest that tonsillectomy may have a therapeutic role in selected patients; however, this hypothesis requires confirmation in larger clinical studies. Mima A et al. presented the case of a 47-year-old Japanese patient with Crohn’s disease who was diagnosed with IgAN after 18 years of infliximab therapy. W leczeniu zastosowano high dose methylprednisolone pulse therapy and oral corticosteroids, without the need for tonsillectomy (Mima et al., 2025).

Baert Ch et al. (Baert et al., 2021) presented a 43-year-old patient suffering from AS. Due to resistance to typical non-steroidal anti-inflammatory drug (NSAID), paracetamol and physiotherapy therapy, INX was included, followed by an improvement in the general condition. Several years later, a deterioration of renal function was incidentally detected, with normal blood pressure. Urinalysis showed proteinuria and haematuria while biopsy showed IgAN. The investigators did not make the connection that the trigger for glomerulopathy could be INX, and included calcium channel blockers (CCBs) and angiotensin-converting enzyme inhibitors (ACEIs). Ultimately, there was an improvement in control of rheumatological and renal symptoms.

### 2.2 Adalimumab

Adalimumab is a full human IgG1 antibody thatbinds to TNF-α and prevents its interaction with TNF receptors. Unlike INX, it is less immunogenic and may therefore be better tolerated in some patients in some patients, and is sometimes used when adverse reactions occur after INX. The most common adverse events include infections (nose and throat, upper respiratory tract infections, sinusitis), injection site reactions (itching, hemorrhage, pain, and swelling), headaches, and musculoskeletal pain.

The available scientific literature describes twelve cases (Premuzic et al., 2020; Strobel et al., 2020; Mertelj et al., 2021; Bhagat Singh et al., 2019; Graziano et al., 2022; Wei and Sinniah, 2013; Pierrot et al., 2018; Shimizu et al., 2024; Tufekci et al., 2026) in which IgAN was diagnosed during adalimumab (ADM) treatment. In one case, hematuria and mild proteinuria were observed before the initiation of treatment (Mertelj et al., 2021). In several cases, renal function was not assessed before the initiation of treatment. Renal function abnormalities, mainly hematuria, proteinuria, increased creatinine, and in some cases hypertension, usually occurred 11 months to 13 years after the initiation of ADM treatment. In each case, a biopsy was performed and IgAN was confirmed, adalimumab was discontinued or replaced with another drug (mainly due to relapse of the underlying disease), mainly infliximab, vedolizumab or rituximab. In one case, there was no improvement after rituximab, therefore baricitinib–JAK (Janus kinase) inhibitor, was introduced, which stabilized renal function but did not resolve the proteinuria (Premuzic et al., 2020). In addition, glucocorticosteroids were used according to the standard treatment regimen, which gave a positive effect. Only in the case of a patient in whom renal dysfunction was noted before the start of treatment, an increased titer of IgA antibodies was noted (Mertelj et al., 2021). In one case, the reaction to ADM discontinuation could not be observed because sepsis occurred and the patient died (Strobel et al., 2020). In the remaining patients, renal function improved and the underlying disease was well controlled. The authors (Wei and Sinniah, 2013) suggested that there is no correlation between the duration of TNFα inhibitor treatment and the severity of glomerular changes, which they attributed to an idiosyncratic response to the drug, which can occur at any time after the initiation of treatment. Tüfekçi, B et al. (Tufekci et al., 2026) described the case of a 44-year-old man diagnosed with seronegative rheumatoid arthritis following a diagnosis of polyarthritis. Adalimumab therapy was initiated in 2014, and in 2019, the patient developed progressive proteinuria. Renal biopsy revealed the presence of IgAN. Suspecting a temporal relationship with adalimumab, the drug was discontinued, which resulted in a significant reduction in proteinuria (to 0.1 g/day) and normalization of serum creatinine levels. Subsequent treatment with tofacitinib did not provide sufficient control of the rheumatoid arthritis. In 2021, certolizumab treatment was initiated, which led to sustained improvement in rheumatoid arthritis symptoms and continued remission of IgAN (proteinuria 0.2 g/day).

### 2.3 Golimumab

Golimumab is a fully humanised IgG1 antibody. Unlike the above, it has higher affinity and greater potency for neutralizing the soluble as well as the membrane-bound form of TNFα. It inhibits the expression of adhesion molecule E-selectin, vascular cell adhesion molecule-1 (VCAM-1) and intercellular adhesion molecule-1 (ICAM-1) of endothelial cells. In addition, it inhibits the secretion of interleukin-6,8 (IL-6,8), granulocyte-macrophage colony-stimulating factor (GM-CSF) induced by TNFα.

Adverse effects of golimumab include upper respiratory tract infections, bacterial, viral, fungal infections, anemia, leukopenia, thrombocytopenia, allergic reactions, depression, insomnia, dizziness and paresthesias. In the first case presented by Lee I.W.Z. et al. (Lee et al., 2024), a 73-year-old Chinese woman with RA who had been in remission for 2 years on golimumab. Baseline creatinine 0.77 mg/dL, eGFR 81 mL/min/1.73 m^2^ with no previous hematuria, proteinuria. She presented with fever and hematuria. On admission, she presented with creatinine 2.75 mg/dL, eGFR 14 mL/min/1.73 m^2^, dysmorphic erythrocytes in urine >2,250/HPF, urine protein/creatinine ratio 2.3 g/g, serum albumin 30 g/L. It was decided to perform a biopsy, which diagnosed IgAN and rapidly progressive glomerulonephritis (RPGN). Golimumab was discontinued, and GCS and mycophenolate mofetil were started. After about 5 months, there was gradual normalization: creatinine 1.23 mg/dL, eGFR 44 mL/min/1.73 m^2^, serum albumin 44 g/L, urine protein/creatinine ratio 0.2 g/g. In the second case (Premuzic et al., 2020), 27-year-old patient with RA, initially treated with methylprednisolone, methotrexate and sulfasalazine, then due to lack of effect, golimumab was included. The patient had no renal impairment before treatment. Prior to treatment. Three years after the inclusion of golimumab, RA remained in remission, but follow-up examinations showed albuminuria, proteinuria, elevated erythrocyte sedimentation rate (ESR). A biopsy performed showed features suggestive of IgAN. Despite this, the decision was made to prolong golimumab therapy by additionally including an ACEI. One year later proteinuria had increased to 3g/day. A decision was made to discontinue golimumab. Prednisone doses were started and cyclophosphamide was switched to azathioprine. Renal function remained stable, while proteinuria decreased. Finally, the patient was redirected to rituximab, azathioprine was stopped, achieving RA clinical remission, proteinuria of 0.09g/day, appropriate renal function.

TNFα inhibitors may affect immune regulation. Changes in T-helper cell responses, including a potential shift from a Th1- toward a Th2-dominant profile and alterations in the production of cytokines such as IL-4, IL-5 and IL-10, have been reported or proposed during TNFα inhibition. However, direct evidence linking these changes to the development of IgAN in patients receiving TNFα inhibitors is lacking. (Montero et al., 2024; Miedziaszczyk et al., 2024). Th2, Th17 and follicular Th-cell responses have been implicated in the immunopathogenesis of IgAN. Patients with IgAN have been reported to exhibit alterations in several T-cell populations, including increased Th2, follicular Th and Th17 cells and reduced Th1 and regulatory T-cell populations. These immune alterations have been associated with increased production of galactose-deficient IgA1 (Gd-IgA1), although their specific contribution to TNFα inhibitor-associated IgAN remains uncertain. (Mertelj et al., 2021). It has been hypothesized that antibodies generated against components of TNFα inhibitors may interact with Gd-IgA1 or related immune components, potentially contributing to immune-complex formation, mesangial deposition and complement activation. However, this proposed pathway has not been directly demonstrated in patients with TNFα inhibitor-associated IgAN (Montero et al., 2024). Some authors have suggested a possible class effect among TNFα inhibitors may exist with regard to IgA glomerulopathy (the exception is etanercept) and therefore a TNFα inhibitor is not recommended in the case of IgAN when it was induced by a drug from the same class. However, the available evidence is limited to case reports, and a class effect cannot currently be considered established (Mertelj et al., 2021). TNFα inhibitors in addition to IgAN can induce: membranous glomerulonephritis (MGI), lupus nephritis, crescentic glomerulonephritis, vasculitis (Segawa et al., 2020). Finally, it is noteworthy that polymorphisms of the TNFα gene have been associated with susceptibility to IgAN in some studies. However, whether such polymorphisms contribute to the risk of drug-associated IgAN remains uncertain (Karras et al., 2015; Syrjanen et al., 2002; Tuglular et al., 2003).

### 2.4 Etanercept

Etanercept is a drug formed by combining two subunits: the extracellular portion of the p75 receptor for TNFα and the Fc fraction of IgG1 immunoglobulin. By binding TNFα molecules, it acts as a fake receptor, which prevents TNFα-dependent cellular response. Moreover, it can modulate the biological response occurring through other molecules such as cytokines. The most common adverse events include injection site reactions, infections, allergic reactions, and fever.

A patient with stage III chronic kidney disease (eGFR 35 mL/min/1,73 m^2^), psoriatic arthritis, hypertension, revascularized coronary artery disease, without proteinuria or hematuria was started on etanercept and after several months of therapy developed proteinuria ≥3.5 g/24 h, treatment was discontinued, and a renal biopsy was performed, which showed IgAN (Nazzaro et al., 2016). Steroid therapy was decided, reducing proteinuria to 1.6g/24 h. In order to further control psoriatic arthritis, adalimumab (anti-TNFα) was included, leading to a re-increase in proteinuria. After 1 year of treatment with cortisone, the arthritis exacerbated, etanercept was switched on again, achieving an increase in proteinuria after several administrations. Treatment was discontinued. After a year, proteinuria recurred again (6.6 g/d), psoriatic pathology exacerbated. Ultimately, the patient is on dialysis, on prednisone maintenance therapy, with no exacerbation of psoriasis. Other cases of IgAN occurring after etanercept use have also been described (Icardi et al., 2003).

Atzeni et al. (Atzeni et al., 2005) suggested that TNFα inhibitors may alter cytokine production and T-helper cell responses, potentially shifting the immune response toward increased production of cytokines associated with Th2 responses. Another proposed mechanism involves reduced CD44 expression and subsequent apoptosis, which could increase the exposure of nuclear antigens and potentially contribute to autoantibody formation. However, these mechanisms remain hypothetical and have not been directly demonstrated as pathways leading to IgAN (Almoallim et al., 2012).

### 2.5 Pembrolizumab and nivolumab

Checkpoint inhibitors (CPIs), specifically anti-programmed cell death protein 1 (PD-1) antibodies, are monoclonal antibodies directed against the PD-1 receptor, present on the surface of activated B lymphocytes, T lymphocytes and natural killer (NK) cells. The physiological function of these receptors is to protect the body from excessive autoimmunity of the immune system, such as after fighting an infection or other cause of inflammation, thereby providing a physiological mechanism of immune downregulation, acting as a physiological immune checkpoint (Asemota et al., 2021). These drugs represent a promising form of treatment forsolid tumors, hematologic malignancies and melanoma (Champiat et al., 2016; Wang et al., 2020). Activation of this receptor suppresses T-cell activity. Cancer cells have the ability to express ligands for the PD-1 receptor (Programmed Death Ligand 1 and 2; PD-L1, PD-L2), thereby silencing cellular immunity. The use of anti-PD-1 antibodies prevents interaction between the receptor and its ligands between the receptor and the ligand of cancer cells, which consequently sustains the anti-tumor activity of the immune system (Wang et al., 2017). The overall incidence of immune-related adverse effects (irAEs) is approximately 26.82% (Asemota et al., 2021), and the most common ones include pruritus, rash, hepatitis, interstitial pneumonia, colitis, thyroiditis and pituitary adenitis. As for CPI nephrotoxicity, it is extremely rare and occurs in the 2%–4.5% range.

When pembrolizumab is used as monotherapy, the incidence of elevated serum creatinine is 1.4% (at any stage of chronic kidney disease; CKD) and 0.2% (in stage 3 or 4 CKD) (Oki et al., 2020). Acute tubulointerstitial nephritis (ATIN) predominates, but membranous nephropathy, minimal change disease, focal segmental glomerular sclerosis and IgA-associated nephropathy have also been described (Wang et al., 2020). Deterioration of renal function associated with nivolumab treatment is estimated at 2% (Puzanov et al., 2017). There have been several reports of patients who developed IgAN after the use of pembrolizumab or nivolumab (Asemota et al., 2021; Wang et al., 2020; Oki et al., 2020; Tanabe et al., 2020; Mamlouk et al., 2019; Mitarai et al., 2022; Kishi et al., 2018; Chabannes et al., 2024; Belkaid et al., 2020). In each case, the drug was discontinued after obtaining biopsy results, which led to resolution of pathomorphological changes. Renal abnormalities resolved after drug discontinuation in most cases, although persistent renal impairment was reported in two patients treated with pembrolizumab (Wang et al., 2020; Tanabe et al., 2020; Chabannes et al., 2024)and one patient treated with nivolumab. Some cases were tested for levels of anti-IgA (Oki et al., 2020; Tanabe et al., 2020), anti-IgE, N-acetyl-β-D-glucosaminidase (NAG) and β2-microglobulin (Mitarai et al., 2022), whose levles increased during disease progression and decreased after drug discontinuation, and sometimes after implementation of systemic GCS therapy.

The mechanism of IgAN is not fully known. Two hypotheses have been proposed: aberrant activation of self-reactive T cells and loss of peripheral tolerance to intrinsic renal antigens (Sprangers et al., 2022). Pembrolizumab has been proposed to promote the formation of antibodies directed against aberrantly glycosylated IgA1, potentially facilitating immune-complex formation and mesangial deposition. This proposed mechanism may contribute to glomerular injury, but it has not been directly demonstrated in patients with pembrolizumab-associated IgAN (Mitarai et al., 2022). It has also been suggested that nivolumab may, in some patients, unmask a previously subclinical form of IgAN through enhanced immune activation; however, this hypothesis remains unproven (Tanabe et al., 2020). In one reported case of IgAN during nivolumab therapy, it was suggested that PD-1 blockade may lead to increasedpathological production of IgA-containing antibodies, either through activation of autoreactive, anti-glycan Th lymphocytes or through re-stimulation of autoreactive memory B cells. This could potentially result in the formation of immune complexes deposited in the glomeruli (Kishi et al., 2018). Ashour et al. suggest that reduced self-tolerance, autoantibody formation, immune complex deposition and immune response may contribute in GN (Ashour et al., 2019).

Oki et al. reported that animal models lacking the gene encoding the PD-1 receptor developed autoimmune diseases and glomerulonephritis with immune complexes. These findings suggest that the PD-1 pathway may contribute to the regulation of T-cell-mediated renal inflammation. Inhibition of the PD-1 pathway may promote hyperreactivity of the immune system, leading to increased aggressiveness of T cells against foreign antigens, as well as impaired recognition and attack of the cells’ own antigens. Such immune dysregulation could potentially contribute to autoimmune or immune-complex-mediated renal injury, although this mechanism has not been directly established in patients with IgAN receiving PD-1 inhibitors. It is thought thatthe gut microbiota, particularly which exhibited qualitative changes and dysbiosis in mice lacking the PD-1 gene, may play a role in the etiology of IgAN. It is noteworthy that animal data suggest that intestinal immune alterations may contribute to IgAN even in the absence of overt intestinal immune-related adverse events such as colitis (Oki et al., 2020). These observations further support the potential involvement of PD-1 signaling in the regulation of systemic immune homeostasis; however, their relevance to human IgAN remains uncertain. Figure 1 presents the targets for biologic drugs modulating the immune response and proposed mechanisms of adverse effects of selected monoclonal antibodies leading to IgAN.

**FIGURE 1:**
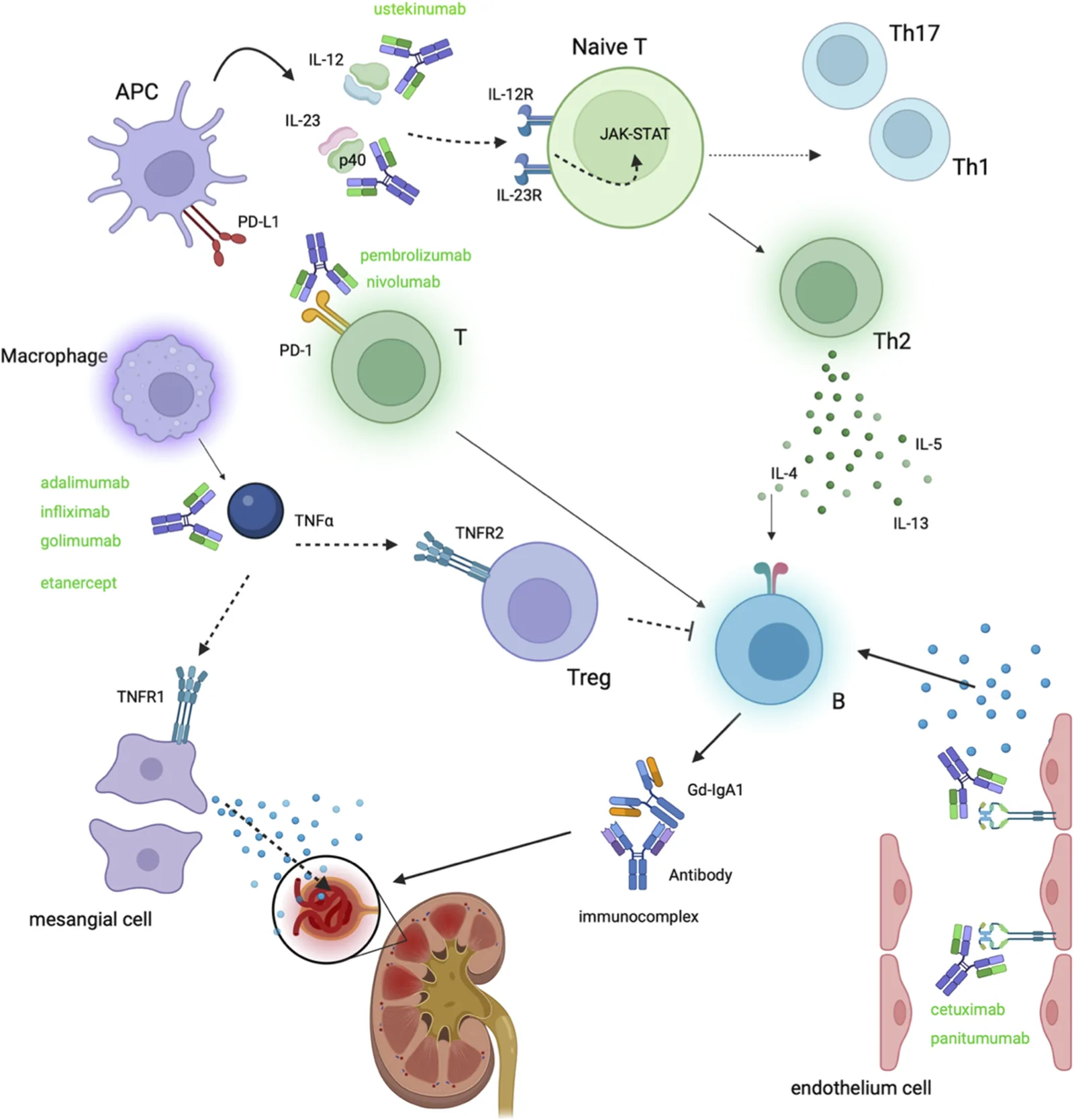
Targets of biologic drugs modulating the immune response and proposed mechanisms potentially contributing to IgA nephropathy associated with selected monoclonal antibodies. Antigen-presenting cells produce the cytokines IL-12 and IL-23, which, via IL-12R and IL-23R receptors on naive T cells, activate the JAK–STAT signaling pathway, leading to T cell differentiation into Th1, Th17, and Th2 subpopulations. Th2 cells may contribute to B-cell activation and production of antibodies, including galactose-deficient IgA1 (Gd-IgA1). Gd-IgA1-containing immune complexes may subsequently deposit in the glomerular mesangium, potentially contributing to mesangial cell activation and renal injury. Macrophages produce TNF-α, which interacts through TNFR1 and TNFR2 receptors, enhancing the inflammatory response. The figure also highlights the targets of selected therapeutic antibodies: ustekinumab (IL-12/23 blockade), pembrolizumab and nivolumab (PD-1 blockade), durvalumab (PD-L1 blockade), TNF-α inhibitors (adalimumab, infliximab, golimumab, etanercept), and anti-EGFR antibodies (cetuximab, panitumumab). Solid lines represent established or well-characterized biological processes, whereas dashed lines represent proposed or hypothetical pathways for which direct mechanistic evidence in monoclonal antibody-associated IgAN is lacking. Legend: APC–antigen-presenting cell; B – B lymphocyte B; IL-12, IL-23 – interleukin; IL-12R, IL-23R–interleukin receptor; p40 – subunit p40 of interleukin 12; PD-1 - programmed death receptor 1; PD-L1 - programmed death-ligand 1; Naive T–naive T cell; T–lymphocyte T cell; Th1, Th2, Th17 – helper lymphocyte T cell; Treg–regulatory T lymphocyte; TNF-α–tumor necrosis factor α; TNFR1, TNFR2 – tumor necrosis factor receptor type 1 or 2.

### 2.6 Ustekinumab

Ustekinumab (UST) targets the P40 subunit of IL-23 and IL-12 and prevents the binding of cytokines to the IL-12R-β1 receptor (Lopez-Ferrer et al., 2017). In addition, it inhibits the production of cutaneous lymphocyte antigen (CLA) acting as a directional target for lymphocytes (Ryan et al., 2010). These cytokines have a distinct function, namely, IL-12 induces the development of Th1 cells producing INF gamma, while IL-23 is involved in the differentiation of Th17 cells acting pro-inflammatory. The products of activated Th17 cells are thought to be important in the pathogenesis of some autoimmune diseases. The use of USTs blocks IL-12/23 signaling and thereby modulates downstream inflammatory responses involved in autoimmune and inflammatory diseases (Lopez-Ferrer et al., 2017). The long-term adverse effects are infections and cardiac disorders (Lopez-Ferrer et al., 2017). Cases of low-grade fever, weight loss, complications of skin cancer, prostate cancer, a possible risk of B-cell lymphoma, epithelioid sarcoma, lung, ovarian, esophageal, testis, kidney and thyroid, Ewing’s sarcoma, pneumonia and reversible posterior leukoencephalopathy syndrome have also been reported (Rao et al., 2021).

The clinical case presented by N. Kanazawa et al. (Kanazawa et al., 2020) describes a 24-year-old man with Crohn’s disease, receiving UST, who developed deterioration of renal function. Renal function tests at the time of diagnosis of Crohn’s disease did not show any abnormalities except for mild proteinuria and hematuria. Initially, the patient was treated with mesalazine and INX, without sufficient therapeutic effect, with deterioration of renal function. The drug was converted to UST, which improved the intestinal symptoms but was followed by deterioration in renal function (albumin 4.5 g/dL, creatinine 2.6 mg/dL, eGFR 27.9 mL/min/1.73 m^2^, CRP <0.1 mg/dL, IgG 1903, mg/dL, IgA 529 mg/dL, IgM 83 mg/dL). Complement levels were within the normal range. Daily urine collection was 0.8 g of proteinuria, while the sediment contained 5–9 erythrocytes per field of view. Urinary markers of tubulointerstitial damage (N-acetyl-β-D-glucosaminidase, β2-microglobulin, α1-microglobulin) were above normal. A kidney biopsy was performed, which revealed crescentic IgAN. UST was withdrawn, oral GCS was administered, and the doses were gradually reduced. In order to protect the residual renal function, mesalazine was discontinued and losartan was introduced. Ultimately, the treatment brought a therapeutic effect confirmed by the improvement of renal parameters, reduction of proteinuria, reduction of IgA and Gd-IgA1 levels in plasma. IgAN may be an extraintestinal manifestation of Crohn’s disease, but the intestinal symptoms disappeared and the deteriorating renal function continued. In the presented case, the fibrocellular crescents occurred, which may suggest that the disease was induced by an unspecified trigger. Histopathological examination of the renal biopsy material revealed tubulointerstitial pathological changes, which are not characteristic of mesalazine (diffuse interstitial nephritis). Another case of nephropathy following UST treatment was reported after UST – 51-year-old man with psoriasis (Perez Fernandez et al., 2019). The patient underwent a renal biopsy, which showed focal segmental glomerulosclerosis of the classic type. UST was discontinued and GCS therapy was initiated, which led to resolution of proteinuria.

Th1, Th17-mediated IL-12/23 and Th2 cells with concomitant IL-4 activation have been implicated in the pathogenesis and progression of Crohn’s disease. A characteristic condition of IgAN is a higher concentration of Th2 than Th1 lymphocytes. IL-4 associated with Th2 lymphocytes enhances Gd-IgA1 formation by altering the terminal glycosylation of secreted IgA1 in vitro models. It has been proposed that ustekinumab may shift the immune response toward Th2 predominance, potentially increasing IL-4 production and Gd-IgA1 synthesis. This remains a hypothetical mechanism that has not been confirmed in clinical studies. (Kanazawa et al., 2020).

### 2.7 Rituximab

Rituximab (RTX) is a chimeric (human-mouse) anti-CD20 monoclonal antibody. It can induces depletion of CD20-positive cells (except plasma cells and stem cells) through complement-dependent cytotoxicity and antibody-dependent cytotoxicity (Cerny et al., 2002; Nishikawa et al., 2024). The main side effects include fever, chills, muscle rigidity, facial flushing, angioedema, bronchospasm, vomiting, nausea, urticaria, symptoms of tumor lysis syndrome.

There is one article in the available literature that indicates the possible occurrence of IgAN after long-term use of RTX. A patient originally suffering from minimal change nephrotic syndrome achieved remission after initial treatment with GCS, cyclosporine and mizoribine. RTX was included to maintain remission. Renal parameters before initiating treatment with monoclonal antibody: creatinine 0.56 mg/dL, IgG 837 mg/dL, serum IgA 364.4 mg/dL, urine protein 0.07g/gCr, no hematuria. Approximately 65–79 months after starting RTX therapy, the following occurred: urine protein level 1.41 g/gCr, latent blood 50–99/HPF, granular shaft 1/HPF, epithelial 2/HPF, fatty 7/HPF, albumin 4.5 g/dL, creatinine 0.97 mg/dL, IgG 185 mg/dL, IgA 552 mg/dL. It was decided to perform a biopsy, which indicated IgAN. The abnormalities subsequently improved following tonsillectomy and courses of GCS and cyclosporine (urine protein level of 0.24 g/gCr, hematuria 5–9/HPF without cellular rollers) (Nishikawa et al., 2024).

The exact mechanism of IgAN induction after RTX is unknown, although tumor necrosis factor (BAFF), proliferation-inducing ligand (April) and B lymphocyte maturation antigen (BCMA) have been proposed as potential contributors. However, this proposed mechanism has not been validated clinically. Given that the available evidence is limited to a case report, a causal relationship between rituximab treatment and the development of IgAN cannot be established (Nishikawa et al., 2024).

Table 1 presents the demographic and clinical data of patients with IgAN associated with the use of monoclonal antibodies.

**TABLE 1:** Demographic and clinical data of patients with IgA nephropathy associated with the use of monoclonal antibodies.

References	Age (sex)	Disease	Monoclonal antibody	Treatment duration	Kidney manifestation	Treatment	Clinical outcome
Jacquet et al., 2009	37(M)	Ankylosing spondylitis	Infliximab	2 years	Hematuria, proteinuria	Withdrawal, ARB	Stable kidney function
Kluger et al., 2015	56(M)	Psoriasis	Infliximab	8 weeks	Hematuria, proteinuria	Withdrawal, ACEI, ustekinumab	Improvement
Segawa et al., 2020	28(F)	Psoriasis	Infliximab	3 years	Hematuria, proteinuria, renal dysfunction	Withdrawal, methylprednisolone pulse therapy, oral corticosteroids, secukinumab	Improvement
Montero et al., 2024	20(M)	Crohn’s disease	Infliximab	7 years	Hematuria, proteinuria, renal dysfunction	Withdrawal, oral corticosteroids, ARB, ustekinumab	Improvement
Shimizu et al., 2024	20s(M) 30s(M) 40s(M)	Crohn’s disease	Infliximab Adalimumab Infliximab	2–6 years	Hematuria, proteinuria, renal dysfunction	Withdrawal, ARB, methylprednisolone pulse therapy, oral corticosteroids, tonsillectomy	Improvement
Mima et al., 2025	47(M)	Crohn’s disease	Infliximab	18 years	Hematuria, proteinuria	Withdrawal, methylprednisolone pulse therapy, oral corticosteroids	Improvement
Baert et al., 2021	43(M)	Ankylosing spondylitis	Infliximab	13 years	Hematuria, proteinuria, renal dysfunction	Withdrawal, ACEI	Improvement
Premuzic et al., 2020	33(M)	Rheumatoid arthritis	Adalimumab	6 years	Proteinuria, hematuria	Withdrawal, increase oral corticosteroids, next rituximab, baricitinib	Stable kidney function
Strobel et al., 2020	19(F) 45(M)	Crohn’s disease	Infliximab Adalimumab	NA	Hematuria, renal dysfunction	Withdrawal, vedolizumab	Improvement
Strobel et al., 2020	74(M)	Crohn’s disease	Adalimumab	2 years	Hematuria, proteinuria, renal dysfunction	Withdrawal	Sepsis, death
Mertelj et al., 2021	33(M)	Crohn’s disease	Adalimumab	7 years	Hematuria, proteinuria, renal dysfunction	Withdrawal, methylprednisolone pulse therapy, oral corticosteroids, ACEI, next vedolizumab	Improvement
Wei and Sinniah, 2013	NA(M)	Psoriasis	Adalimumab	18 months	Hematuria, proteinuria, renal dysfunction	Withdrawal, oral corticosteroids	Improvement
Pierrot et al., 2018	45(M)	Rheumatoid arthritis	Adalimumab	NA	Proteinuria	Withdrawal, oral corticosteroids, tocilizumab	Improvement
Graziano et al., 2022	11(M)	Crohn’s disease	Adalimumab	2 years	Hematuria, renal dysfunction	Withdrawal, oral corticosteroids, ACEI	Improvement
Bhagat Singh et al., 2019	39(F)	Crohn’s disease	Adalimumab	4 years	Hematuria, renal dysfunction	Withdrawal, infliximab	Improvement
Tufekci et al., 2026	44(M)	Seronegative rheumatoid arthritis	Adalimumab	5 years	Hematuria, proteinuria	Withdrawal, tofacitinib, certolizumab	Improvement
Lee et al., 2024	73(F)	Rheumatoid arthritis	Golimumab	2 years	Proteinuria, hematuria, renal dysfunction	Withdrawal, methylprednisolone pulse therapy, oral corticosteroids, MMF	Marked improvement
Premuzic et al., 2020	27(F)	Rheumatoid arthritis	Golimumab	4 years	Proteinuria	Oral corticosteroids, cyclophosphamide, next azathioprine, rituximab	Remission
Nazzaro et al., 2016	NA	Psoriatic arthritis	Etanercept and adalimumab	NA	Proteinuria, renal dysfunction	Withdrawal, corticosteroids	renal dysfunction progression, HD
Wang et al., 2020	72(M)	Epitheiloid mesothelioma of the left lung	Pembrolizumab	5 months	Renal dysfunction	Withdrawal for 2 months, next pembrolizumab	Persistent renal impairment
Oki et al., 2020	75(F)	Non-small cell lung cancer (NSCLC)	Pembrolizumab	9 months	Hematuria, proteinuria	Withdrawal	Improvement, NSCLC relapse
Tanabe et al., 2020	78(M)	Advanced gastric cancer	Nivolumab	2 months	Hematuria, proteinuria	Withdrawal, oral corticosteroids	Improvement, tumor regrowth
Mitarai et al., 2022	70(M)	Squamous cell lung cancer	Pembrolizumab	7 courses	Hematuria, proteinuria, renal dysfunction	Withdrawal, methylprednisolone pulse therapy, oral corticosteroids	Improvement proteinuria, persistent renal impairment
Chabannes et al., 2024	65(M)	NSCLC	Pembrolizumab	9 weeks	Hematuria, renal dysfunction	Withdrawal, cyclophosphamide, oral corticosteroids	Tumor regrowth, multiple-organ failure, death
Kishi et al., 2018	72(M)	Squamous cell lung cancer	Nivolumab	10 months	Hematuria, proteinuria, renal dysfunction	Withdrawal	Improvement
Belkaid et al., 2020	70(M)	Melanoma	Nivolumab	4 months	Hematuria, proteinuria, renal dysfunction	Withdrawal, methylprednisolone pulse therapy, oral corticosteroids	Improvement
Kanazawa et al., 2020	24(M)	Crohn’s disease	Ustekinumab	3 months	Hematuria, proteinuria, renal dysfunction	Withdrawal, oral corticosteroids, ARB	Improvement

Legend: ACEI, angiotensin-converting enzyme inhibitor; ARB, angiotensin II, receptor blockers, F–female, HD, hemodialysis, M–male, MMF, mycophenolate mofetil; RPGN, rapidly progressive glomerulonephritis.

## 3 Monoclonal antibodies associated with the risk of other glomerulonephritis than IgAN

### 3.1 Membranous nephropathy

Membranous nephropathy is an autoimmune disease characterized by the deposition of immune complexes along the glomerular capillary walls, leading to thickening of the glomerular basement membrane and impaired glomerular function (Ronco et al., 2021; Gu et al., 2021; Couser, 2017). In most cases, no underlying cause can be identified, however there have been cases of patients developing membranous nephropathy secondary to treatment with alemtuzumab (Alchi and Jayne, 2010).

Alemtuzumab is a recombinant humanized monoclonal antibody against CD52 glycoprotein (Rabant et al., 2021; Syed, 2021). CD52 is abundantly expressed on the surface of T lymphocytes and B lymphocytes, whereas lower levels of expression are found on natural killer cells, monocytes, and macrophages.

Alemtuzumab significantly reduces the population of T and B lymphocytes, which is used in the treatment of relapsing-remitting multiple sclerosis (Floisand et al., 2019). So far, 3 cases of membranous nephropathy have been reported in the treatment of alemtuzumab in patients with relapsing-remitting multiple sclerosis (patients A, B and C). The patients presented with nephritic syndrome, while serum creatinine levels remained within the normal range at 12 months (patient A), 5 months (patient B) and 13 months (patient C), respectively. In patients B and C, the diagnosis of membranous nephropathy was confirmed by a kidney biopsy, and in patient A, glomerular IgG deposits were detected in immunofluorescence. Patients B and C were started on ACE inhibitors and diuretics (Rabant et al., 2021; Phelps et al., 2019). Patient B achieved partial remission with GFR within the normal range and minimal proteinuria, while patient C achieved complete remission of the disease (Phelps et al., 2019). No data were available on further treatment or its effects in patient A.

A case of membranous nephropathy was also reported in a 61-year-old woman with rheumatoid arthritis receiving adalimumab, a monoclonal anti-TNF-α antibody (Maruotti et al., 2009). The patient developed membranous nephropathy accompanied by nephrotic syndrome during adalimumab therapy. Although the temporal relationship between adalimumab administration and the onset of renal disease raises the possibility of a drug-related mechanism, a direct causal relationship could not be definitively established. This case, together with other reports of glomerular disease occurring during anti-TNF-α treatment, suggests that monoclonal TNF-α inhibitors may rarely be associated with immune-mediated renal injury.

Another case of membranous nephropathy associated with monoclonal anti-TNF-α therapy was reported in a 29-year-old woman with severe psoriasis treated with infliximab (Chin et al., 2005). The patient received four infusions of infliximab and developed nephrotic syndrome approximately 1 month after the last dose. Clinical manifestations included generalized peripheral edema, ascites, pleural effusion, and periorbital swelling. Laboratory investigations revealed nephrotic-range proteinuria of approximately 13 g/day, severe hypoalbuminemia (10 g/L), and hypercholesterolemia, while serum creatinine remained within the normal range. Serological investigations, including antinuclear antibodies, hepatitis B and C, and HIV testing, were negative. Renal biopsy confirmed membranous nephropathy, demonstrating granular IgG and C3 deposition along the glomerular capillary walls and subepithelial electron-dense deposits on electron microscopy. As the patient had no other apparent cause of secondary membranous nephropathy and was not receiving concomitant medication, the authors considered infliximab to be the probable cause of the renal disease.

Membranous nephropathy has also been reported in a patient treated with durvalumab for non-small cell lung cancer (Kim, 2021). Durvalumab is a human IgG monoclonal antibodyagainst programmed death ligand 1 (PD-L1), therebythereby preventing PD-L1 from interacting with PD-1 and CD80. Thereby enhancing immune-mediated recognition and destruction of cancer cells (Antonia et al., 2017). In the case of the described patient, it is important to emphasize that 8 years before the diagnosis of non-small cell lung cancer, he developed nephritic syndrome. Following the administration of durvalumab, the patient developed anasarca after 9 months. Laboratory tests showed a decrease in albumin levels, increased LDL cholesterol, creatinine concentration within the normal range (0.98 mg/dL) and protein content in a 24-h urine collection of 11,230 mg. A kidney biopsy showed accumulations of eosinophils and thickening of the glomerular basement membrane (Kim, 2021). In the described patient, it is postulated that the recurrence of membranous nephropathy may have been associated with durvalumab treatment, potentially through enhanced immune activation. However, the temporal relationship alone does not establish a causal relationship (Kim, 2021).

A case of membranous nephropathy was also described as a consequence of treatment with Certolizumab pegol (CZP). CZP is a PEGylated, Fab'-only, recombinant humanized monoclonal antibody that binds and neutralizes TNFα (Lee and Scott, 2020; Dhillon, 2014). The drug is approved in the European Union for the treatment of patients with severe active axial spondyloarthritis, including ankylosing spondylitis and non-radiographic axSpA, as well as active psoriasis (Dhillon, 2014). According to the literature, a possible mechanism of renal pathology during treatment with TNFα antagonists is immune dysregulation. Anti-nuclear antibodies, anti-double stranded DNA antibodies and anticardiolipin antibodies may develop during treatment (Stokes et al., 2005). Bilateral lower extremity pitting edema, hypoalbuminemia and proteinuria were observed in a 65-year-old woman 6 months after starting treatment with cerolizumab for rheumatoid arthritis (Butendieck et al., 2016). Additional examinations did not reveal any abnormalities, including double-stranded DNA (dsDNA). Certolizumab treatment was discontinued after a total of 12 months of therapy, when the patient’s kidney biopsy confirmed the diagnosis of membranous glomerulonephritis. Steroids were not used in further therapy, but torsemide and losartan. After 25 months from the discontinuation of certolizumab pegol, the patient had only trace proteinuria and mild peripheral edema (Butendieck et al., 2016).

A case of membranous nephropathy has also been reported in a patient treated with denosumab (Kimoto et al., 2020). Denosumab is a fully human monoclonal antibody that binds to and inhibits receptor activator of nuclear factor kappa-B ligand (RANKL), thereby preventing its interaction with the RANK receptor. The drug is used primarily in the treatment of osteoporosis and in the prevention of skeletal-related complications in patients with bone metastases. RANKL and RANK are also expressed in renal tissue, including podocytes, which has led to the hypothesis that the RANKL/RANK pathway may have a role in maintaining glomerular function. The relevance of this pathway to denosumab-associated membranous nephropathy, however, remains uncertain.

The described patient was a 67-year-old woman with rheumatoid arthritis and osteoporosis who developed proteinuria after initiation of denosumab therapy. Before treatment, renal function was normal and there was no evidence of proteinuria. Approximately 3 months after the first administration of denosumab, proteinuria was detected and subsequently increased to approximately 2.8 g/g creatinine. A kidney biopsy performed 6 months after the initiation of denosumab showed findings consistent with membranous nephropathy, including thickening of the glomerular basement membranes and granular deposition of IgG and C3 along the glomerular capillary walls. Electron microscopy demonstrated subepithelial and intramembranous electron-dense deposits. PLA2R staining was negative. Other potential causes of secondary membranous nephropathy, including malignancy and infection, were not identified. Although the patient had a history of rheumatoid arthritis, the disease had remained stable and there was no evidence of renal involvement before denosumab treatment. Denosumab was discontinued after the diagnosis of membranous nephropathy, and no immunosuppressive therapy was administered. Following withdrawal of the drug, proteinuria gradually decreased and almost complete remission was achieved approximately 2.5 years later, with urinary protein excretion decreasing to 0.1 g/g creatinine. The temporal association between denosumab administration and the onset of proteinuria, the absence of other identifiable causes of secondary membranous nephropathy, and the subsequent improvement after discontinuation of treatment suggest a possible association between denosumab and membranous nephropathy.

### 3.2 Focal segmental glomerulosclerosis

Focal segmental glomerulosclerosis (FSGS) is the leading glomerular cause of end-stage renal disease in the United States. Depending on the etiology of the disease, primary and adaptive FSGS are mainly distinguished. The presumed cause of primary FSGS is a circulating permeability factor, and primary FSGS is characterized by responsiveness to immunosuppressive therapy and a risk of recurrence after kidney transplantation, while excessive nephron workload due to increased body size, reduced nephron capacity or single glomerular hyperfiltration caused by certain diseases are responsible for the adaptive form (Rosenberg and Kopp, 2017).

Focal segmental glomerulosclerosis has been reported during certolizumab pegol treatment; however, the available evidence is limited to individual case reports and does not establish causality. A case was reported of a 51-year-old patient with Crohn’s disease who suffered from joint pain that did not respond to other medications. Six months after treatment initiationwith CZP, the patient began to complain of foaming urine and increasing edema of the limbs and face. Further examination showed proteinuria (10 g/day) and an increase in creatinine level to 4 mg/dL. Kidney biopsy was performed and the diagnosis of focal and segmental glomerulosclerosis was made. The patient was started on steroids and diuretics, resulting in gradual improvement in renal function (Leong and Fung-Liu, 2010). Figure 2 shows the most common types of glomerulonephritis after the use of monoclonal antibodies.

**FIGURE 2:**
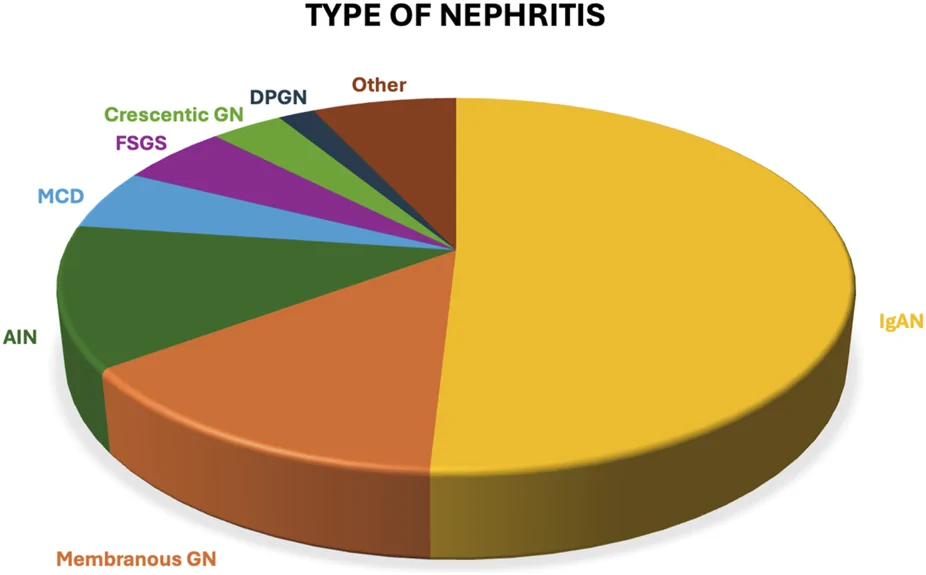
Frequency summary of reports in the available scientific literature on nephritis following the use of monoclonal antibodies. Legend: Crescentic GN- crescentic glomerulonephritis; DPGN–diffuse proliferative glomerulonephritis; FSGS–focal segmental glomerulonephritis; IgAN–IgA nephropathy; MCD–minimal change disease; Membranous GN–membranous glomerulonephritis; MPGN - membranoproliferative glomerulonephritis.

Ranibizumab is a recombinant humanized anti-vascular endothelial growth factor A (VEGF-A) monoclonal antibody fragment. This prevents VEGF-A from binding to its receptors, thereby inhibiting endothelial proliferation, vascular permeability, and neovascularization. It has six times higher affinity than bevacizumab (Cai and Bressler, 2017). Because of its mechanism of action, ranibizumab is used, among other indications, in ophthalmology for the treatment of macular edema. In a diabetic patient (T2DM) with CKD stage 5, after initiation of intravitreal anti-VEGF therapy of anti-VEGF drugs because of vitreous hemorrhage, significant deterioration of renal function was noted (Shye et al., 2020). The patient was initially treated with bevacizumab, which was replaced with ranibizumab due to worsening macular edema. Shortly after starting anti-vascular endothelial growth factor (VEGF) therapy, the patient’s proteinuria and renal function deteriorated, and his creatinine level reached 10.5 mg/dL, requiring renal replacement therapy. The patient did not consent to a kidney biopsy, which made it impossible to make a clear diagnosis (Shye et al., 2020).

Bevacizumab is a humanized monoclonal antibody directed against VEGF-A. Binding of bevacizumab to VEGF-A prevents VEGF-A from interacting with its receptors, thereby inhibiting proliferation and neovascularization. Bevacizumab is used, among others, in ophthalmology. Several cases of bevacizumab-associated nephrotoxicity have been described of bevacizumab nephrotoxicity described in the available medical literature. A 59-year-old patient with poorly controlled diabetes mellitus who developed significant deterioration of renal function after intravitreal injection of bevacizumab (total dose 16.25 mg). His albuminuria and serum creatinine levels increased significantly, while eGFR decreased to 36 ml/min from a baseline of 73 ml/min. The final diagnoses were diffuse and nodular diabetic glomerulosclerosis and segmental glomerulosclerosis with focal collapsing features and interstitial nephritis. Further deterioration of the patient’s kidney function required the initiation of hemodialysis treatment (Shye et al., 2020). Another patient who also experienced deterioration of kidney function after treatment with anti-VEGF drugs is a 46-year-old patient suffering from diabetes. The patient was started on intravitreal injections of bevacizumab at a total dose of 7.5 mg, and then switched to ranibizumab (0.6 mg total dose). Renal function deteriorated, albuminuria increased, eGFR decreased and serum creatinine increased. Based on the performed kidney biopsy, the diagnosis of diffuse and nodular diabetic glomerulosclerosis and likely drug/medication-induced interstitial nephritis was made. The patient’s kidney function continued to deteriorate, which led to the initiation of renal replacement therapy with hemodialysis (Shye et al., 2020).

In addition, another case is known of a 41-year-old patient with T2DM and CKD stage 3 who was also treated with intravitreal injections of bevacizumab every 6 weeks for a year. The patient reported to the hospital with dyspnea lasting for a month and with pitting edema of the lower limbs for a year. Serum creatinine was slightly elevated (2.15 mg/dL) and the patient’s urinalysis showed proteinuria, microscopic hematuria, and granular casts. A kidney biopsy was performed, whose findings were consistent with diabetic nephropathy class 3 by Tervaert classification. The patient’s creatinine remained elevated. ACEI/ARB treatment was not started due to decreased glomerular filtration rate. As the disease progressed, the patient was treated with renal replacement therapy (Ahmed et al., 2021). The presented clinical cases show that kidney damage during anti-VEGF treatment does not have a clear pathomorphological picture.

### 3.3 Minimal change disease

Minimal change disease (MCD) has been reported as a rare renal complication of monoclonal antibody therapy, with the most clearly documented cases involving bevacizumab. The available literature identifies three cases of MCD associated with bevacizumab, with an important distinction between the route of administration. The two intravitreal cases were reported by Sato et al. (Sato et al., 2013) and Perez-Valdivia et al. (Perez-Valdivia et al., 2014) In both reports, intravitreal bevacizumab was associated with a relapse of previously diagnosed MCD, rather than the development of *de novo* disease. Thus, these cases suggest that systemic exposure following local ocular administration may be sufficient to trigger recurrence of an underlying podocytopathy in susceptible patients. Both patients were treated with systemic corticosteroids. In contrast, Hanna et al. (Hanna et al., 2016) described the first reported *de novo* case of biopsy-proven MCD following systemic intraveonus bevacizumab administration in an adult. The patient was a 72-year-old man with anaplastic astrocytoma who developed marked proteinuria approximately 4 weeks after initiation of bevacizumab administered every 2 weeks. He subsequently developed nephrotic syndrome and progressive acute kidney injury, with the serum creatinine increasing from a baseline of 0.8 mg/dL to a peak of 4.8 mg/dL and urinary protein excretion reaching approximately 19.5 g/g creatinine. Renal biopsy demonstrated complete podocyte foot-process effacement on electron microscopy, with no immune deposits and no evidence of focal and segmental glomerulosclerosis, supporting the diagnosis of MCD. Importantly, withdrawal of bevacizumab alone did not result in clinical improvement; renal function continued to deteriorate for more than 2 weeks after discontinuation. High-dose prednisone was subsequently initiated, resulting in rapid improvement in renal function and gradual resolution of proteinuria.

The intravitreal cases demonstrate that ocular administration cannot be considered entirely devoid of systemic renal effects, despite the local route of administration. However, the available reports do not allow a direct comparison of the incidence or relative risk of MCD between intravitreal and IV bevacizumab, because these observations are based exclusively on isolated case reports rather than comparative studies or systematic pharmacovigilance data.

The distinction is particularly important because bevacizumab-associated renal toxicity is not limited to MCD. Other reported glomerular lesions following IV administration include collapsing glomerulonephritis and membranoproliferative glomerulonephritis. Thus, the development of proteinuria during bevacizumab therapy does not necessarily indicate MCD and, in patients with persistent or nephrotic-range proteinuria, renal biopsy may be required to establish the underlying lesion. MCD has also been reported in association with other monoclonal antibodies. The review by Hanna et al. cites one case of MCD associated with IV trastuzumab, in addition to reports of focal and segmental glomerulosclerosis and fibrillary glomerulonephritis following trastuzumab therapy. Overall, the published cases comprise four reported MCD events associated with monoclonal antibodies in the cited compilation: three related to bevacizumab (two intravitreal and one IV) and one related to IV trastuzumab.

### 3.4 Diffuse proliferative glomerulonephritis

Diffuse proliferative glomerulonephritis (DPGN) is an inflammatory process characterized by involvement of at least 50% of the glomeruli and increased cell proliferation (Ikhlas and Anjum, 2026). This type of glomerulonephritis may occur in the course of many pathological processes, andhas also been reported during treatment with some biopharmaceuticals. To date, one case of diffuse proliferative glomerulonephritis has been reported in patients treated with cetuximab (Manthri et al., 2017).

Cetuximab is a recombinant chimeric monoclonal antibody against epidermal growth factor receptor (EGFR) and which is used in the treatment of patients with locally advanced, recurrent or metastatic squamous cell carcinoma of the head and neck (SCCHN) and metastatic colorectal cancer (mCRC) (Wong, 2005; Azzoli et al., 2009). So far, only three cases of glomerular injury after administration of cetuximab have been reported in the literature and one of which involved diffuse proliferative glomerulonephritis (Azzoli et al., 2009; Sasaki et al., 2013; Koizumi et al., 2017). Other renal lesions reported during cetuximab treatment includedwith Cetuximab were IgA-dominant proliferative with crescentic glomerulonephritis and pathology with features of thrombotic microangiopathy (TMA). In both of the last described patients, kidney function improved after discontinuation of cetuximab treatment, while in the first one suffering from DPGN it was necessary to start hemodialysis (Manthri et al., 2017; Sasaki et al., 2013; Koizumi et al., 2017).

### 3.5 Immune complex membranoproliferative glomerulonephritis

Panitumumab is a recombinant fully human IgG2 monoclonal antibody that binds to the extracellular domain III of the epidermal growth factor receptor (Stremitzer et al., 2014). This prevents ligand-induced autophosphorylation of the receptor, it prevents autophosphorylation of the receptor induced by its ligands (Brazilian Medical et al., 2018). This results in receptor internalization of the receptor, inhibition of cell growth, induction of apoptosis, as well as a decrease in the secretion of pro-inflammatory interleukin 8 and vascular endothelial growth factor (Brazilian Medical et al., 2018). Panitumumab has an affinity approximately eight times higher than that of cetuximab than that of another EGFR-targeting biologic–cetuximab. Panitumumab is an FDA approved monotherapy for refractory metastatic colorectal cancer (Stremitzer et al., 2014). A case of a patient receiving panitumumab for metastatic colorectal cancer was reported. After two doses of this drug, the patient developed nephrotic syndrome, proteinuria (6 g/day) and decreased serum albumin (2 g/dL). The patient underwent a kidney biopsy, which showed enlarged glomeruli with slightly thickened mesangium, thrombi in the capillary lumen, and capillary wall and mesangial deposits of IgA, IgG, and C1q. Cryoglobulinic GN was excluded due to undetectable levels of serum cryoglobulins. Based onthese findings, it has been suggested that immuno-complex glomerulonephritismay have been associated with panitumumab treatment. However, given that the evidence is derived from a single case, a causal relationship cannot be established. Panitumumab was discontinued in the patient and steroid therapy with prednisolone was started. After 4 weeks, the proteinuria significantly decreased, the albumin level increased to normal, and the creatinine concentration was then 49 μmol/L (Izzedine et al., 2017).

### 3.6 Rapidly progressive glomerulonephritis (crescentic GN)

Crescentic glomerulonephritis, also known as rapidly progressive glomerulonephritis, can result from many different disease processes. Regardless of the cause, crescentic glomerulonephritis is characterized by rapid deterioration of renal function, and crescents are found on light microscopy of the kidney biopsy. They are multilayered accumulations of proliferating cells in the Bowman’s membrane (Wang et al., 2020). Crescentic glomerulonephritis has been reported in association with treatment with biologic agents, including certolizumab pegol, although the available evidence does not establish a causal relationship.

Certolizumab pegol (CZP) is a PEGylated, Fab’ only, recombinant humanized monoclonal antibody that binds and neutralizes soluble and membrane-bound tumor necrosis factor α (TNFα) (Lee and Scott, 2020; Dhillon, 2014). The drug is registered in the European Union for the treatment of patients with severe active axial spondyloarthritis, including ankylosing spondylitis and non-radiographic axSpA, as well as active psoriasis (Dhillon, 2014). According to the literature, a possible mechanism of renal pathology during treatment with TNFα antagonists is immune dysregulation. Anti-nuclear antibodies, anti-double stranded DNA antibodies and anticardiolipin antibodies may develop in the body during treatment (Stokes et al., 2005). The available literature describes a case of a 30-year-old woman treated for seropositive rheumatoid arthritis, successfully treated with methotrexate, who discontinued methotrexate therapy due to her desire to become pregnant. Certolizumab pegol was included as an alternative treatment. Six months after initiation of biological treatment, laboratory testing revealed an increase in serum creatinine (which increased from0.52 mg/dL to 1.27 mg/dL within 1 month), proteinuria (1.3 g/day), microscopic hematuria and pathological casts (Funada et al., 2021). Autoantibody testing revealed myeloperoxidase anti-neutrophil cytoplasmic autoantibodies (MPO-ANCA). Renal function did not improve significantly after discontinuation of CZP. Based on the kidney biopsy findings and the presence of MPO-ANCA, the patient was diagnosed with microscopic polyangiitis (MPA) with MPO-ANCA-associated rapidly progressive glomerulonephritis and necrotizing vasculitis. The temporal association with certolizumab treatment raises the possibility of a drug-associated immune-mediated process; however, causality cannot be definitively established from this case alone (Funada et al., 2021).

An 87-year-old man with hypertension and rheumatoid arthritis, who had been treated with golimumab for 4 years, was admitted because of worsening renal function (creatinine 2.6 mg/dL; previously 1.29 and 0.9 mg/dL). Urinalysis revealed significant proteinuria and hematuria with acanthocytes, and serological tests were negative. The clinical and laboratory findings suggested necrotizing crescentic GN. During hospitalization, renal function remained stable (2.5–2.8 mg/dL). Golimumab was discontinued, and high-dose prednisone (60 mg) and rituximab were initiated. After 1 month, renal function improved and proteinuria decreased; after 6 months, renal parameters significantly improved (creatinine 1.4 mg/dL). The authors used the Naranjo scale to assess the likelihood that the renal adverse event was drug-related. A score of 6 indicates a probable adverse drug reaction (Agrawal et al., 2021).

## 4 Acute interstitial nephritis

Acute interstitial nephritis (AIN) is a common cause of acute kidney injury. Currently, the most common cause of AIN is the use of drugs, especially from the group of non-steroidal anti-inflammatory drugs, antimicrobials and proton pump inhibitors (Yang et al., 2020; Praga and Gonzalez, 2010). The disease is caused by an immune reaction against endogenous nephrogenic antigens or exogenous antigens processed by tubular cells. In the renal parenchyma, infiltrates from immune cells begin to appear (cell-mediated immunity plays a key role in pathogenesis), which are the source of cytokines that increase the production of extracellular matrix and the number of interstitial fibroblasts. As a consequence, interstitial fibrosis then occurs (Praga and Gonzalez, 2010). One of the drugs that can cause AIN is the biologic Omalizumab. Omalizumab is a recombinant human IgG1 monoclonal antibody that binds to free and membrane-bound IgE antibodies, thereby reducing total free IgE but not the receptor-bound IgE (El-Qutob, 2016). It is approved for the treatment of severe allergic asthma and chronic urticaria. A case of a 71-year-old patient was described, in which, after the administration of the second dose of omalizumab, the general condition deteriorated and chronic diseases exacerbated. The performed exams revealed creatinine of 714.27 μmol/L, blood urea nitrogen of 49.62 mmol/L and urine microalbumin/creatinine ratio of 2,524 mg/g. The patient required starting renal replacement therapy. The biopsy revealed moderate diffuse glomerulosclerosis, focal segmental glomerulosclerosis, and moderate arterio- and arteriolosclerosis. Eosinophils near the corticospinal junction have also been noted. After 8 weeks of steroid therapy, the patient recovered from acute kidney injury (AKI) and did not require continued renal replacement therapy (Yang et al., 2020).

Cases of acute interstitial nephritis have also been reported in Vedolizumab therapy (Bailly et al., 2018; Simpson et al., 2021; Floisand et al., 2019). Vedolizumab is a monoclonal antibody directed against the α4β7 integrin that mediates migration of lymphocytes to the gastrointestinal mucosa. Thanks to this, the drug found a use in the treatment of inflammatory bowel diseases, including ulcerative colitis (CU) and Crohn’s disease (Floisand et al., 2019). Among the two patients treated with vedolizumab (patient X due to CU, patient Y due to Crohn’s disease renal function deteriorated at various times since the last dose of the biologic. In one of them (Y) the signs of kidney damage appeared several hours after administration of the drug, while in the other (X) only after a week. Creatinine increased to 410 μmol/L and 171 μmol/L, respectively. In both patients a kidney biopsy was performed, which confirmed the diagnosis of acute interstitial nephritis. These patients were then treated with systemic steroid therapy, thanks to which, after 1 month (patient Y) and 3 weeks (patient X) of treatment, normal kidney function was achieved in both patients (Bailly et al., 2018; Subhaharan et al., 2022). Considering the exhaustion of all therapeutic options in the treatment of a patient with Crohn’s disease, it was decided to continue treatment with vedolizumab, this time simultaneously with glucocorticosteroids. No adverse renal effects were noted after any of the subsequent infusions (Subhaharan et al., 2022).

## 5 Renal cortex necrosis

Renal failure associated with cortical necrosis has also been reported among the complications of antibody treatment. Renal cortical necrosis was registered in therapy with alemtuzumab in a 39-year-old female patient with multiple sclerosis (Liou et al., 2019). 4 h after alemtuzumab administration, the patient’s condition began to deteriorate rapidly. Eight hours after drug administration, the patient developed thrombocytopenia, anemia, and acute kidney injury. 3 days after exposure to the medicine, due to hypervolemia and associated respiratory failure, the patient required hemodialysis treatment. Immunoassays did not show the presence of anti-glomerular basement membrane antibodies. A laparoscopic biopsy of the left kidney performed after 10 days of hospitalization revealed cortical necrosis. As a result of 8-month of renal replacement therapy, the patient managed to obtain a normal platelet count (after just 7 weeks), a normal red blood cell count and a reduction of creatinine levels to the normal range. In the described case, alemtuzumab caused drug-induced thrombotic microangiopathy (Liou et al., 2019). Table 2 presented drugs associated with a risk of nephropathy.

**TABLE 2:** Drugs associated with the risk of nephropathy.

International name	Brand name	Target	US approval year	First indication	Type of glomerulonephritis	Supporting references(s)
Infliximab	Remicade	TNFα	1998 (FDA) 1999 (EMA)	Crohn disease	IgAN, FSGS	Jacquet et al., 2009, Segawa et al., 2020, Shimizu et al., 2024, Kluger et al., 2015; Montero et al., 2024, Mima et al., 2025, Baert et al., 2021
Etanercept	Enbrel	TNFα	1998 (FDA) 2000 (EMA)	Rheumatoid arthritis	IgAN	Icardi et al., 2003; Nazzaro et al., 2016
Adalimumab	Humira	TNFα	2002 (FDA) 2003 (EMA)	Rheumatoid arthritis	IgAN	Wei and Sinniah, 2013; Pierrot et al., 2018; Bhagat Singh et al., 2019; Strobel et al., 2020; Mertelj et al., 2021; Graziano et al., 2022; Shimizu et al., 2024; Tufekci et al., 2026
Certolizumab pegol	Cimzia	TNFα	2008 (FDA), 2009 (EMA)	Rheumatoid arthritis	FSGS, membranous nephropathy, crescentic glomerulonephritis	Leong and Fung-Liu, 2010
Golimumab	Simponi	TNFα	2009 (FDA, EMA)	Rheumatoid arthritis, psoriatic arthritis, ankylosing spondylitis	IgAN, crescentic glomerulonephritis	Lee et al., 2024
Pembrolizumab	Keytruda	PD1	2014 (FDA), 2015 (EMA)	Advanced melanoma	IgAN	Mitarai et al., 2022; Gallan et al., 2021; Ashour et al., 2019
Nivolumab	Opdivo	PD1	2014 (FDA), 2015 (EMA)	Advanced melanoma	IgAN	Kishi et al., 2018; Asemota et al., 2021; Gallan et al., 2021; Ashour et al., 2019
Durwalumab	Infinzi	PDL1 (PD1, CD80)	2017 (FDA), 2018 (EMA)	Non-small cell lung cancer (NSCLC)	Membranous nephropathy	Kim, 2021; Gallan et al., 2021
Cetuximab	Erbitux	EGFR	2004 (FDA, EMA)	Colorectal cancer (metastatic, EGFR-positive)	IgAN, DPGN, crescentic glomerulonephritis	Gallan et al., 2021; Manthri et al., 2017; Manthri et al., 2017
Panitumumab	Vectibix	EGFR	2006 (FDA), 2007 (EMA)	Metastatic colorectal cancer (EGFR-positive)	Immune-complex glomerulonephritis	Izzedine et al., 2017
Ranibizumab	Lucentis	VEGF-A	2006 (FDA), 2007 (EMA)	Neovascular form of age-related macular degeneration (AMD)	AIN	Shye et al., 2020
Bevacizumab	Avastin	VEGF-A	2004 (FDA), 2005 (EMA)	Colorectal cancer	AIN	Shye et al., 2020
Rituximab	MabThera (EU), Rituxan (US)	CD20	1997 (FDA) 1998 (EMA)	Non-Hodgkin B-cell lymphoma	IgAN	Nishikawa et al., 2024
Alemtuzumab	Campath	CD52	2001 (FDA, EMA)	B-cell chronic lymphocytic leukemia	IgAN, membranous nephropathy, renal cortex necrosis	Phelps et al., 2019; Rabant et al., 2021, Liou et al., 2019
Omalizumab	Xolair	IgE	2003 (FDA), 2005 (EMA)	Severe, chronic asthma	AIN	Yang et al., 2020
Ustekinumab	Stelara	IL-12/23	2009 (FDA, EMA)	Plaque psoriasis	IgAN	Kanazawa et al., 2020
Vedolizumab	Entyvio	Integrin α4β7	2014 (FDA, EMA)	Crohn disease, ulcerative colitis	AIN	Bailly et al., 2018; Simpson et al., 2021; Subhaharan et al., 2022

Legend: CD20 – B-lymphocyte antigen CD20, TNFα, tumor necrosis factor α, CD52 – cluster of differentiation 52, IgE–immunoglobulin E, EGFR, epidermal growth factor receptor; VEGF-A, vascular endothelial growth factor, IL12/IL23 – interleukin 12/interleukin 23, integrin α4β7 – lymphocyte Peyer’s patch adhesion molecule-1, PD-1, programmed death receptor 1; PD-L1, programmed death-ligand 1, CD80 – cluster of differentiation 80, IgAN, IgA nephropathy; FSGS, focal segmental glomerulosclerosis; AIN, acute interstitial nephritis; DPGN, diffuse proliferative glomerulonephritis.

## 6 Conclusion

The level of evidence for drug associated glomerular disease associated with monoclonal antibodies is generally limited. The available literature consists primarily of case reports and small case series, with very few observational studies. We have presented all published reports suggesting a possible association between monoclonal antibodies and IgA nephropathy and other nephropathies, while clearly acknowledging that in most cases the evidence is limited to case reports or case series and does not confirm or not exclude a causal relationship.

In patients treated with anti-TNFα drugs, renal function and urinalysis should be monitored before and during therapy with these drugs. When renal dysfunction or abnormalities on laboratory or biopsy tests occurred during the course of biologic treatment, treatment teams mainly discontinued the agent suspected of causing the disorder, and sometimes additionally included GCS to reduce proteinuria.

The occurrence of IgAN can have a negative impact on the underlying disease, as in the case of nivolumab in the treatment of NSCLC, when GCS was used as a symptomatic treatment, leading to a shorter progression-free time, in a mechanism of the effect of immunosuppression on Non-Small Cell Lung Cancer (NSCLC) progression.

A case report by Rikako Oki et al. cites pharmaceutical recommendations for pembrolizumab, according to which if creatinine levels rise to grade 2 (1.5–3x baseline), the drug should be withheld, while for grade 3 (>3x baseline), steroid treatment such as prednisolone at 1–2 mg/kg is recommended. International clinical guidelines recommend corticosteroids as first-line treatment for moderate to severe immune-related adverse events (irAEs). However, the use of corticosteroids requires consideration in the case of anticancer monoclonal antibodies, whether it will have a progressive effect on the underlying disease. Although studies have shown that GCS use is associated with better long-term renal function preservation compared with no GCS use with pembrolizumab (Mitarai et al., 2022), glucocorticoid-induced immunosuppression may impair antitumor immune responses and potentially affect tumor control.

From a clinical perspective, before initiating monoclonal antibody therapy, particularly in patients with pre-existing renal disease, an assessment of renal function, should be considered. This assessment should include serum creatinine, estimated glomerular filtration rate (eGFR), urinalysis, and quantification of proteinuria. Periodic monitoring during treatment may facilitate early detection of renal damage. In patients who develop persistent hematuria, proteinuria, or unexplained deterioration in renal function, nephrology consultation should be considered. Decisions regarding discontinuation of therapy in the event of suspected monoclonal antibody involvement should be made on an individual basis, taking into account the severity of renal damage and the benefit of treating the underlying disease. A practical approach to the management of renal adverse events after their occurrence during mAb therapy, according to the severity of kidney injury based on the CTCAE and KDIGO classifications, is summarized in Table 3. Given that the current evidence is derived predominantly from case reports and small case series, these recommendations should be regarded as pragmatic clinical considerations rather than formal evidence-based guidelines.

**TABLE 3:** Practical recommendations for the assessment and management of renal adverse events during monoclonal antibody therapy, informed by the Common Terminology Criteria for Adverse Events classification (CTCAE) and Kidney Disease Improving Global Outcomes KDIGO criteria.

Clinical stage (eGFR range)	Renal assessment	CTCAE description	Clinical procedure	Treatment	Kidney biopsy
Before mAb therapy (NA)	Creatinine, eGFR, urinalysis, UACR/UPCR, BP; assess CKD and renal risk factors. Frequency: first month every week, then every 3 months	​	Establish baseline; review concomitant nephrotoxic drugs	Optimize BP and volume status; avoid nephrotoxins where possible	Not indicated routinely
Grade 1/mild abnormality (60–89)	Repeat creatinine/eGFR and urinalysis; assess proteinuria every 7–14 days	Asymptomatic; laboratory findings only; proteinuria (0,3–1 g/24 h)	Continue mAb if clinically appropriate; closer monitoring; exclude reversible causes	Supportive treatment (ACEI/ARB)	Observation, usually not indicated
Grade 2/moderate renal toxicity (30–59)	Repeat laboratory assessment within days (every 1–7 days); quantify proteinuria; assess urinary sediment	Edema; hypertension; oral management indicated	Consider temporary interruption of mAb; nephrology consultation; exclude alternative causes	Supportive treatment (ACEI/ARB), consider immunosupresive drugs after establishing diagnosis (kidney biopsy)	Consider
Grade 3/severe renal toxicity (15–29)	Urgent renal assessment; monitor creatinine, eGFR, urine output, electrolytes	Hospitalization or prolongation of existing hospitalization indicated; limiting self-care activities of daily living	Immediate discontinuation of mAb, urgent nephrology consultation	Supportive treatment (ACEI/ARB), immunosupresive treatment according to diagnosis (biopsy result)	Recommended
Grade 4/life-threatening (requires RRT)	Urgent assessment including indications for RRT	Life-threatening consequences; urgent intervention indicated; RRT	Immediate discontinuation of mAb, urgent nephrology consultation	RRT when indicated, immunosupresive drugs, immunosupresive treatment according to diagnosis (biopsy result)	Recommended -individualized according to clinical condition

Legend: eGFR, estimated glomerular filtration rate; ACEI, angiotensin-converting-enzyme inhibitors, ARB, angiotensin receptor blockers; RBC-HPF, red blood cells per high power field; RBC, red blood cells; RRT, renal replacement therapy, mAb–monoclonal antibodie.

This review has several limitations. First, it was designed as a narrative review rather than a systematic review. This approach was intentionally chosen because the currently available evidence on monoclonal antibody-associated IgAN is limited predominantly to individual case reports and small case series, with substantial heterogeneity in patient characteristics, implicated agents, clinical presentation, diagnostic work-up, and treatment strategies. The narrative format allowed us to integrate the published clinical observations with current knowledge of the potential pathogenic mechanisms and clinical implications of this rare adverse event. Nevertheless, the conclusions presented in this review should be interpreted with caution, and further multicenter studies and larger observational cohorts are needed to better define the incidence, pathogenesis, and optimal management of monoclonal antibody-induced IgA nephropathy.

An important limitation of this article is confounding by indication. Several underlying diseases treated with monoclonal antibodies, including inflammatory bowel disease, rheumatoid arthritis, psoriasis, and other chronic inflammatory disorders, have themselves been associated with glomerular diseases, particularly IgAN. Consequently, distinguishing between drug-induced nephropathy and renal involvement related to the underlying disease is often difficult, especially in reports lacking pre-treatment renal evaluation or long-term follow-up. Therefore, the temporal relationship between monoclonal antibody therapy and glomerular disease should be interpreted with caution.

In addition to TNF inhibitors, other monoclonal antibodies are responsible for the occurrence of glomerulonephritis. When starting therapy with monoclonal antibodies, one should be aware of possible renal complications.

## Glossary

ACEI: Angiotensin-Converting Enzyme Inhibitor
ADM: Adalimumab
AIN: Acute Interstitial Nephritis
AKI: Acute Kidney Injury
APRIL: A Proliferation-Inducing Ligand
ARB: Angiotensin II Receptor Blocker
ATIN: Acute Tubulointerstitial Nephritis
BAFF: B-Cell Activating Factor
BCMA: B-Cell Maturation Antigen
CCB: Calcium Channel Blocker
CKD: Chronic Kidney Disease
CLA: Cutaneous Lymphocyte Antigen
CU: Ulcerative Colitis
CZP: Certolizumab Pegol
DPGN: Diffuse Proliferative Glomerulonephritis
dsDNA: Double-Stranded DNA
EGFR: Epidermal Growth Factor Receptor
eGFR: Estimated Glomerular Filtration Rate
ESR: Erythrocyte Sedimentation Rate
FcγR: Fc Gamma Receptor
FcγRIIIb: Fc Gamma Receptor IIIb
FSGS: Focal Segmental Glomerulosclerosis
GCS: Glucocorticosteroids
Gd-IgA1: Galactose-Deficient IgA1
GM-CSF: Granulocyte-Macrophage Colony-Stimulating Factor
ICAM-1: Intercellular Adhesion Molecule 1
IgAN: IgA Nephropathy
IL: Interleukin
INX: Infliximab
irAE: Immune-Related Adverse Event
JAK: Janus Kinase
mCRC: Metastatic Colorectal Cancer
MGN: Membranous Glomerulonephritis
MPA: Microscopic Polyangiitis
**MPGN**–: Membranoproliferative Glomerulonephritis
MPO-ANCA: Myeloperoxidase Anti-Neutrophil Cytoplasmic Antibody
NAG: N-Acetyl-β-D-Glucosaminidase
NK: Natural Killer Cells
NSCLC: Non-Small Cell Lung Cancer
NSAID: Non-Steroidal Anti-Inflammatory Drug
PD-1: Programmed Death 1
PD-L1: Programmed Death Ligand 1
PD-L2: Programmed Death Ligand 2
RPGN: Rapidly Progressive Glomerulonephritis
RTX: Rituximab
SCCHN: Squamous Cell Carcinoma of the Head and Neck
T2DM: Type 2 Diabetes Mellitus
Th: T Helper cell
TMA: Thrombotic Microangiopathy
TNF-α: Tumor Necrosis Factor Alpha
Treg: Regulatory T Cells
UST: Ustekinumab
VCAM-1: Vascular Cell Adhesion Molecule 1
VEGF: Vascular Endothelial Growth Factor
VEGF-A: Vascular Endothelial Growth Factor A
